# Optimisation of the Mechanical Properties and Mix Proportion of Multiscale-Fibre-Reinforced Engineered Cementitious Composites

**DOI:** 10.3390/polym15173531

**Published:** 2023-08-24

**Authors:** Bowei Yang, Chen Wang, Song Chen, Kaixin Qiu, Jiuhong Jiang

**Affiliations:** 1 College of Civil Engineering, Architecture and Environment, Hubei University of Technology, Wuhan 430068, China; 102110969@hbut.edu.cn (B.Y.); 102010862@hbut.edu.cn (C.W.); 102010881@hbut.edu.cn (S.C.); 102110917@hbut.edu.cn (K.Q.); 2 China Construction Seventh Engineering Division Co., Ltd., Zhengzhou 450003, China; 3 China Construction Third Engineering Bureau (Shenzhen) Co., Ltd., Shenzhen 518000, China

**Keywords:** ECC, PE, CWs, CNTs, response surface method

## Abstract

Engineered cementitious composites (ECCs) are cement-based composite materials with strain-hardening and multiple-cracking characteristics. ECCs have multiscale defects, including nanoscale hydrated silicate gels, micron-scale capillary pores, and millimetre-scale cracks. By using millimetre-scale polyethylene (PE) fibres, microscale calcium carbonate whiskers (CWs), and nanoscale carbon nanotubes (CNTs) as exo-doped fibres, a multiscale enhancement system was formed, and the effects of multiscale fibres on the mechanical properties of ECCs were tested. The Box-Behnken experimental design method, which is a response surface methodology, was used to construct a quadratic polynomial regression equation to optimise ECC design and provide an optimisation of ECC mix proportions. The results of this study showed that a multiscale reinforcement system consisting of PE fibres, CWs, and CNTs enhanced the mechanical properties of ECCs. CWs had the greatest effect on the compressive strengths of highly ductile-fibre-reinforced cementitious composites, followed by CNTs and PE fibres. PE fibres had the greatest effect on the flexural and tensile strengths of high-ductility fibre-reinforced cementitious composites, followed by CWs and CNTs. The final optimisation results showed that when the ECC matrix was doped with 1.55% PE fibres, 2.17% CWs, and 0.154% CNTs, the compressive strength, flexural strength, and tensile strength of the matrix were optimal.

## 1. Introduction

Concrete has the advantages of high compressive strength, low cost, and easy availability of materials, and it has become a widely used building material. However, due to the brittle failure, low tensile strength, and poor crack resistance of concrete, its application in engineering is limited. To improve the above shortcomings of concrete, researchers incorporated various fibres into concrete to made fibre-reinforced concrete (FRC). Although FRC mixed with steel fibre, carbon fibre, glass fibre, and so on improves the toughness of concrete to a certain extent, the strain-softening phenomenon shown in the tensile process causes limited improvement in the ultimate tensile strength and tensile strain [1]. To improve the performance of FRC and meet the demand for FRC with strain-hardening ability, Naaman et al. [2] conducted a lot of research on the damage morphology of the matrix. It was found that the matrix damage morphology depends on the fibre length, aspect ratio, volume fraction, spatial distribution, and pullout behaviour of the fibre, as well as the properties of the matrix. This also prompted researchers to come to a new understanding of FRC. Based on the previous research results, in 1987, Naaman [3] first defined the conditions leading to strain-hardening behaviour and multiseam cracking of FRC in tension, which provided an important basis for subsequent research. Based on a micromechanical model, Li [4] proposed the design concept of FRC with strain-hardening characteristics by reasonably controlling the properties of the fibre and the matrix and the interface parameters of fibre/matrix. On this basis, an ECC with higher tensile strength and tensile strain was successfully prepared [5,6,7,8]. ECCs are prepared using cement mortar as the matrix, fine quartz sand as the aggregate, and a certain amount of soft fibre (usually PVA fibre or PE fibre). The ultimate tensile strains of ECCs are 3~12%, which are several hundred times those of ordinary cementitious materials; additionally, the damage shows multiple obvious cracking patterns, and the cracks can all be maintained at approximately 60 μm [9,10,11]. It can be concluded that ECCs have good toughness and meet the requirements of complex toughness, crack resistance, and durability.

PE fibres have high tensile strength and modulus of elasticity values and are suitable for acidic, alkaline, and high-temperature environments, making them particularly useful for producing ECCs. Li [5], based on the theory of ECC preparation, selected PE fibre to prepare a PE-fibre-reinforced ECC with excellent flexural toughness and flexural properties.

Wang [12] investigated the effects of PE fibres with different volume fractions (0%, 1%, 1.5%, and 2%) on the macroscopic and microscopic properties of ECCs. When the PE fibre content in the ECC reaches 1%, the ECC shows strain hardening and multiple seam cracking, and on the microscopic level, as the PE fibre content increases, the bridging properties of its ECC fibre and matrix show a trend of first increasing and then decreasing.

Liu [13] conducted mechanical property tests on high- and early-strength cementitious composites (HE-ECCs) mixed with steel–PE fibres and investigated the effects of ultrafine fly ash and rubber particles on the properties. The test results show that partial replacement of PE fibres with steel fibres or ultrafine fly ash can improve the compressive strength and tensile modulus values of HE-ECC mixes. In terms of microstructure, ultrafine fly ash significantly improves the fracture toughness and physical adhesion strength between the PE fibres and the matrix.

Xia [14] investigated the effects of four different fine aggregates on the mechanical properties of HE-ECCs. The results showed that the different types of fine aggregates have little effect on the fluidity of PE-ECCs but have a relatively great effect on the compressive strength, uniaxial tensile strength, and ultimate tensile strain.

Yi [15] found that the slip-hardening parameter (β) of ultrahigh-strength ultrahigh-ductility cementitious composites (UHS-UHDCCs) is closely related to the inclination angle of PE fibres, and an increase in the inclination angle of PE fibres leads to an increase in β. A fine-scale mechanical model of single-PE-fibre pullout has been proposed and defined as physical debonding. The results show that the pullout load-displacement curve and single-crack stress–tension curve of a single PE fibre verify the accuracy of the proposed UHS-UHDCC fine-scale mechanical model.

A foundation of ECC research is the micromechanical model. The micromechanical model of ECCs is focused on microstructural and microscopic phenomena [5,16]. The damage of cement matrix composites starts from the microscopic scale [17,18,19], where many cracks are generated in the microstructure; stress concentration occurs at the cracks. These small and irregular cracks develop and accumulate rapidly, and when the cracks reach saturation, macroscopic main cracks can be observed; eventually, the main cracks crack completely, leading to the destruction of the material. The fibres commonly used are in millimetre sizes; thus, the fibres have difficulty addressing the microscopic-size shortcomings of concrete. However, with the gradual maturity of micromaterial and nanomaterial research, the application of micromaterials and nanomaterials blended with macroscopic fibres in cementitious composites can solve this problem [20,21,22,23].

Cao [24] added microscale CWs to cementitious composites and concluded that the resulting strengthening of the microstructure can inhibit the formation of microcracks, improve the fine pore structure, reduce the brittleness of cementitious materials, and improve their micromechanical properties.

Pan [25] designed multiscale fibre-reinforced cementitious composites with PVA fibres and CWs; the experimental results show that for the ECC, the compressive strength is significantly increased and that the tensile strain hardening behaviour is improved when the CW volume fraction is 6%. Microstructural analysis shows that CWs and PVA fibres have good interactions at different sizes.

Muradyan [26] studied the effects of different CNTs concentration (0.001, 0.01, 0.05, and 0.1 wt.%) on the compressive strengths of cement mortars. The results show that the 7 d strengths of the specimens increase by 13% and 19.5% with and without surfactant, respectively, and that the 28 d compressive strengths increase by 6.3% and 13.8%, respectively.

Liu [27] incorporated 0%, 0.05%, 0.08%, 0.10%, 0.15%, 0.20%, and 0.30% mass replacement proportions of MWCNTs at water–cement ratios of 0.25, 0.3, 0.35, and 0.4. The experimental results show that under a fixed water–cement ratio, with increasing CNT doping, the compressive and flexural strengths of the cementitious composites first increase and then decrease. The bridging and filling effects of CNTs are observed by electron microscopy.

Chaipanich [28] used scanning electron microscopy (SEM) and X-ray diffraction (XRD) analyses to study the microstructures and morphologies of carbon nanotube cement composites. The scanning electron microscopy images show good interactions between carbon nanotubes and the cement matrix. CNTs act as fine aggregates at the microscale/nanoscale, making their microstructures increasingly dense and robust. The microstructures and mechanical properties of the cement matrix composites are improved after the incorporation of carbon nanotubes.

Lin [29] fabricated carbon-fibre-reinforced polymer (CFRP) matrix composite laminates containing conductive nanofillers composed of different mass fractions of carbon black (CB), CNTs, and mixed CB and CNTs. The lightning damage tolerance levels of CFRP matrix composite laminates containing conductive nanofillers were evaluated using simulated lightning tests. The results show that the addition of CB and CNTs can reduce the lightning damage degree of CFRP composites while damaging the bending performance.

Lampkin [30] chose to use CNTs as an additive to create a conductive resin matrix to improve the conductivity, thereby reducing the damage and increasing the residual strength of the material. The results show that due to the limited size of the manufactured CFRP plate, the residual strength of the CFRP composite material under the lightning test is not ideal, but the addition of CNTs effectively improves the conductivity of the CFRP composite material and reduces the damage of the lightning strike to the CFRP composite material.

Wu [31,32] used hybrids of CWs and partial substitutions of PE fibres to study their effects on the tensile and compressive properties of engineering cement-based composites. Cao [33] formed a new hybrid fibre system by adding steel fibres, PVA, and CWs and evaluated the flexural toughness. Li [34] used PVA fibres, CNTs, and granulated blast furnace slag to modify the properties of ECCs. However, the above studies involve little research on three different scales of fibres and do not feature comparisons of the effects of three different fibres on the mechanical properties of ECCs; in addition, an optimised design scheme for the comprehensive performance of the experimental design is not provided. Therefore, we propose the construction of a multiscale fibre reinforcement system using nanoscale CNTs, microscale CWs, and millimetre-scale PE fibres to control the defects at different levels through multilevel fibres to generally improve the mechanical properties of highly ductile fibre-reinforced cementitious composites. In this paper, we use nanoscale CNTs, microscale CWs, and millimetre-scale PE fibres as external doping fibres to design the effects of single-factor doping on the strengths of ECC specimens with three different scales of fibres; additionally, we use the Box-Behnken regression model in Design Expert software to establish a regression model with compressive strength, flexural strength, and tensile strength serving as response values. Compressive strength, flexural strength and tensile strength are used as response values to analyse the interactions between the two factors on the response values; the prediction results are given to derive the optimal ratio that meets the response value requirements, which provides a reference for the multiobjective optimisation of the ECC mix proportion.

## 2. Materials and Methods

### 2.1. Test Materials and Parameters

The cement was Conch PO42.5-grade silicate cement with a density of 3155 kg/m^3^ and a specific surface area of 3600 m^2^/kg. In the test performed at the college, the first-grade fly ash used was 2.55 g/cm^3^ with a stacking density of 1.12 g/cm^3^ and a bulk density of 1.12 g/cm^3^. ISO-standard sand was used for fine aggregates. The chemical compositions of cement and fly ash are shown in Table 1.

**Figure 1:**
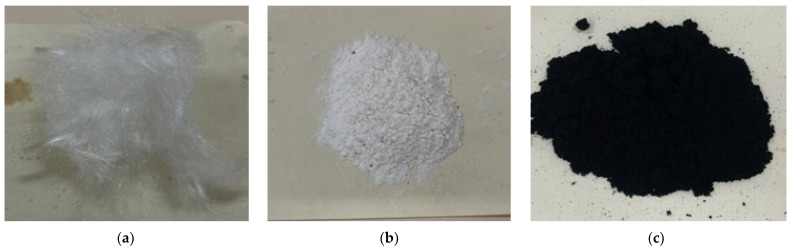
Appearance of PE (**a**), CWs (**b**), and CNTs (**c**).

PE fibre is an excellent insulating material with high strength, low density, excellent chemical and heat resistance, and exceptional mechanical properties, and these properties make it widely applied in various industries. In this paper, we used domestic PE fibres with specific performance indices, as shown in Table 2, and morphologies, as shown in Figure 1. CWs show excellent dispersion and enhancement characteristics in cement mortar, which could significantly improve the tensile strength and compressive strength and effectively control the shrinkage and crack generation of cement mortar, thus improving the overall mechanical properties and service life. We used CWs produced by Jinan Quanxin Chemical Co. The specific performance index is shown in Table 2, and the morphology is shown in Figure 1. The CNTs were multiwalled and visible to the naked eye as a black powder, and they were easily entangled by the extremely small CNTs under conventional conditions and required special surface treatment and dispersion conditions before use [35,36,37]; additionally, they were dispersed through the external doping of a PVP surface activator and the ultrasonic dispersion method.

### 2.2. Specimen Preparation

1. The standard sand and all cementitious materials (cement, fly ash, and CWs) were poured into the mixer and dry-mixed for 3 min at a low speed for even mixing.
2. Water-reducing agent solution and dispersed carbon nanotube solution were simultaneously added. Then, low-speed mixing was performed for 3 min, followed by 1 min of high-speed mixing, to obtain a mixed and stirred slurry of all cementitious materials.
3. PE fibres were added within 1 min of each other at a low speed, after which the mixer was adjusted to a high speed for five minutes.
4. The mixed-cement-based mixture was vibrated in the mould and smoothed.
5. After waiting for 24 h, the mould was removed. Subsequently, the formed test block was placed in the curing room. The curing room conditions were a temperature of 20 °C ± 2 °C, a relative humidity of 95% ± 5%, and a curing time of 28 days.

### 2.3. Test Mix Design

#### 2.3.1. Single-Factor Experiment

The water–cement ratio was 0.3 and the sand–cement ratio was 0.5. Fly ash replaced 50% of the mass of cement, and the water reducing agent was mixed with 1% of the mass of cementitious material. We investigated the effects of PE fibres at mass replacement proportions of 0, 0.5%, 1%, 1.5%, and 2.0% on the mechanical properties of ECCs; these specimens had 2% volume ratios of CWs and 0.10% mass ratios of CNTs. We investigated the effects of 0, 1%, 2%, 3%, and 4% mass replacement proportions of CWs on the mechanical properties of ECCs; these specimens had 1.5% volume ratios of PE fibres and 0.10% mass ratios of CNTs. The effects of 0, 0.05%, 0.1%, 0.15%, and 0.2% mass replacement proportions of CNTs on the mechanical properties of ECCs were investigated; these specimens had 1.5% volume ratios of PE fibres and 2% volume ratios of CWs. The single-factor experiment mix proportion is shown in Table 3.

#### 2.3.2. Response Surface Test

Based on the results of single-factor experiments, the mechanical properties of compressive strength, ultimate tensile stress, and ultimate tensile strain were used as response values, and three factors were selected as independent variables for PE fibres (Factor A), CWs (Factor B), and CNTs (Factor C). A three-factor, three-level experimental design was conducted using Design-Expert 13 software, and the test factors and levels are shown in Table 4.

### 2.4. Mechanical Performance Experiments

#### 2.4.1. Compression and Flexural Test

**Figure 2:**
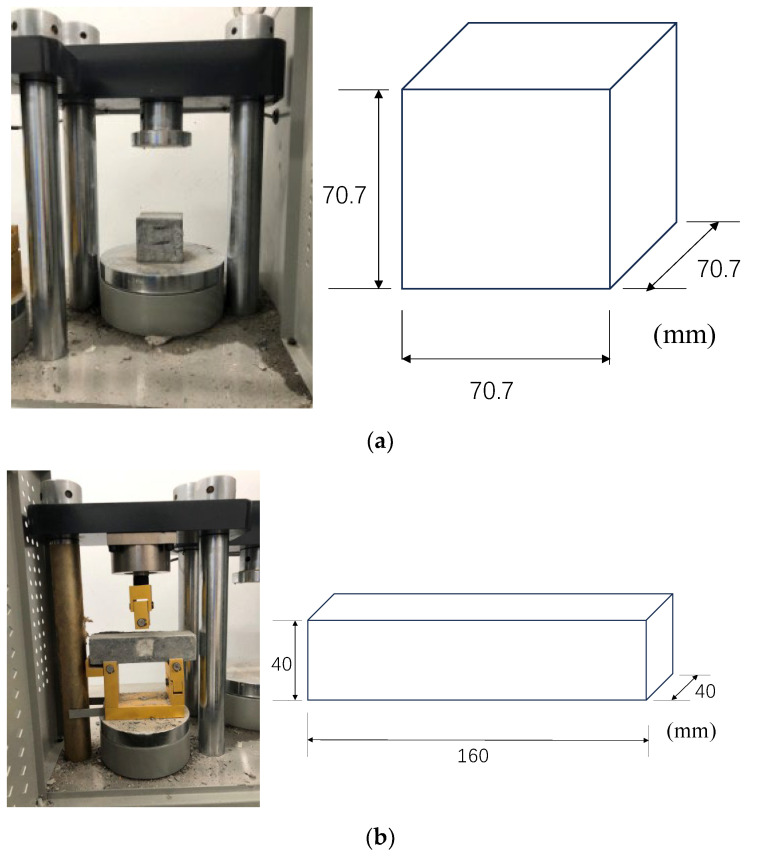
Employed compression test (**a**) and flexural test (**b**) in this study. (**a**) Compression test and dimensions of specimens. (**b**) Flexural test and dimensions of specimens.

Compression and bending performance tests were carried out on a model DYE-300s microcomputer servo cement bending and compression testing machine, as shown in Figure 2. The test procedure for the compressive performance test was carried out according to the steps described in the test method for the basic properties of construction mortar (JGJ/T70-2009) [38]. The compressive specimen had dimensions of 70.7 mm × 70.7 mm × 70.7 mm and a cubic shape, the loading method of the equipment was load control, and the loading rate was 50 N/s. The flexural specimen was a 160 mm × 40 mm × 40 mm cuboid, and the test procedure of the flexural performance test method was the ISO standard cementitious sand strength test [39].

#### 2.4.2. Axial Tensile Test

**Figure 3:**
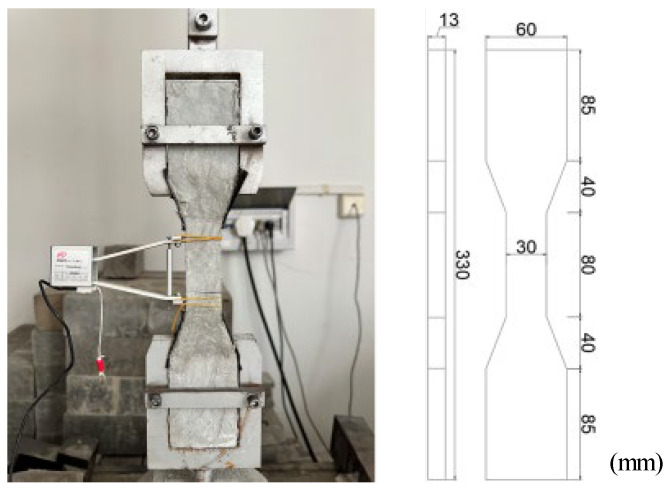
Tensile test block.

An axial tensile performance test was conducted on top of a model WDW-100CDE universal testing machine, as shown in Figure 3. The specimen had a dog-bone shape, which was recommended by the Japanese Society of Civil Engineers (JSCE 2008) [40]. The specific dimensions are shown in Figure 3. On the side of the tensile specimen, a strain displacement sensor was installed to measure the elongation. The equipment loading method was used for displacement control, and the loading rate was constant at 0.2 mm/min.

### 2.5. Microscopic Experiments

After the axial tensile test, a representative specimen of each type of fibre was selected for scanning electron microscopy (SEM) observation and analysis. Due to the good ductility of the PE-ECC, the specimens at the end of the tensile test became integral; additionally, a blade was used to cut parts of them at their main cracks for SEM testing. A ZEISS GeminiSEM 300 was used to photograph the specimen morphology.

## 3. Results and Discussion

### 3.1. Single-Factor Impact Analysis

**Figure 4:**
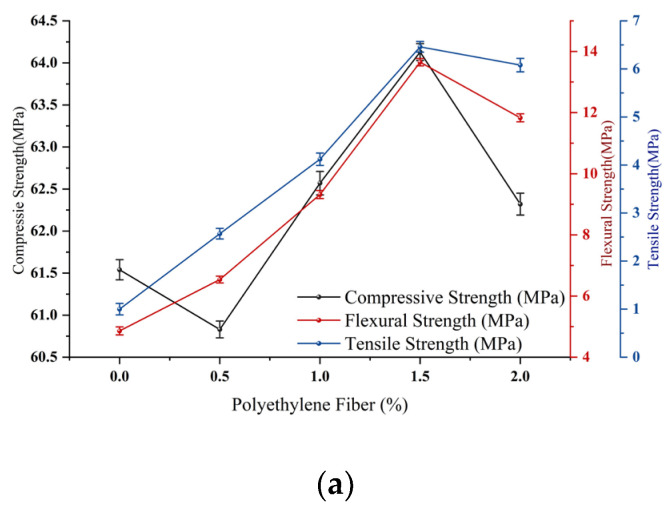

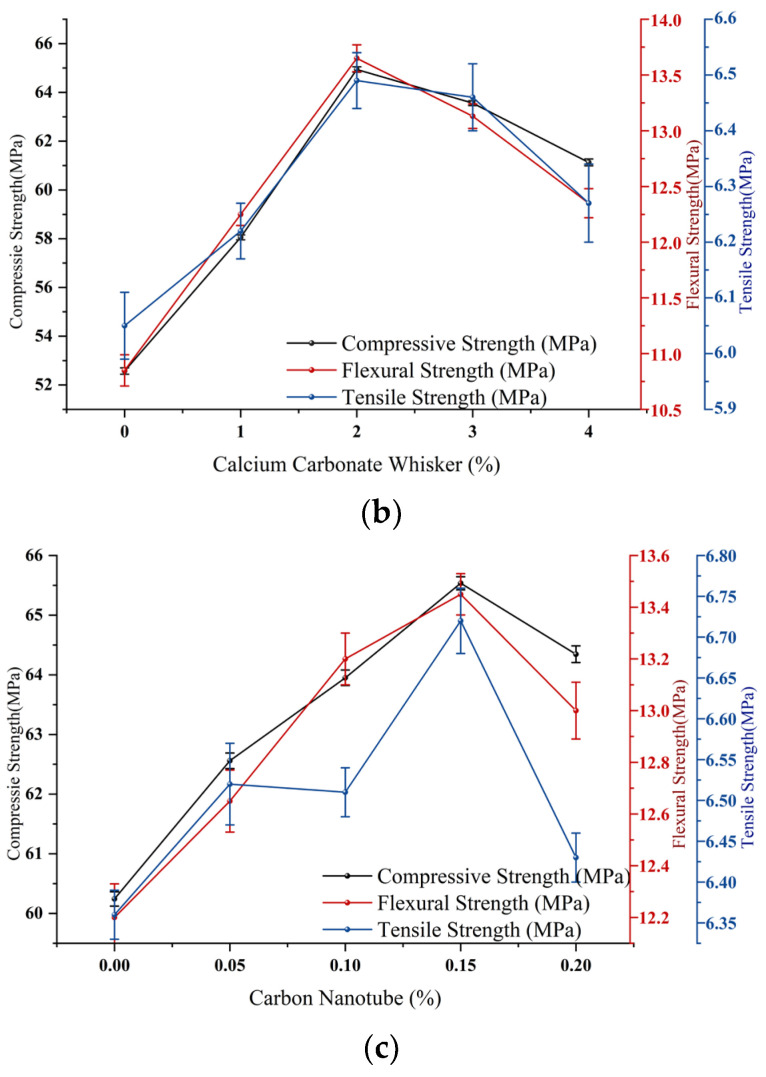
Univariate effects of PE (**a**), CWs (**b**), and CNTs (**c**). (**a**) The influence of PE fibre. (**b**) The influence of CWs. (**c**) The influence of CNTs.

The effects of each factor on compressive strength, flexural strength, and tensile strength are shown in Figure 4.

**Figure 5:**
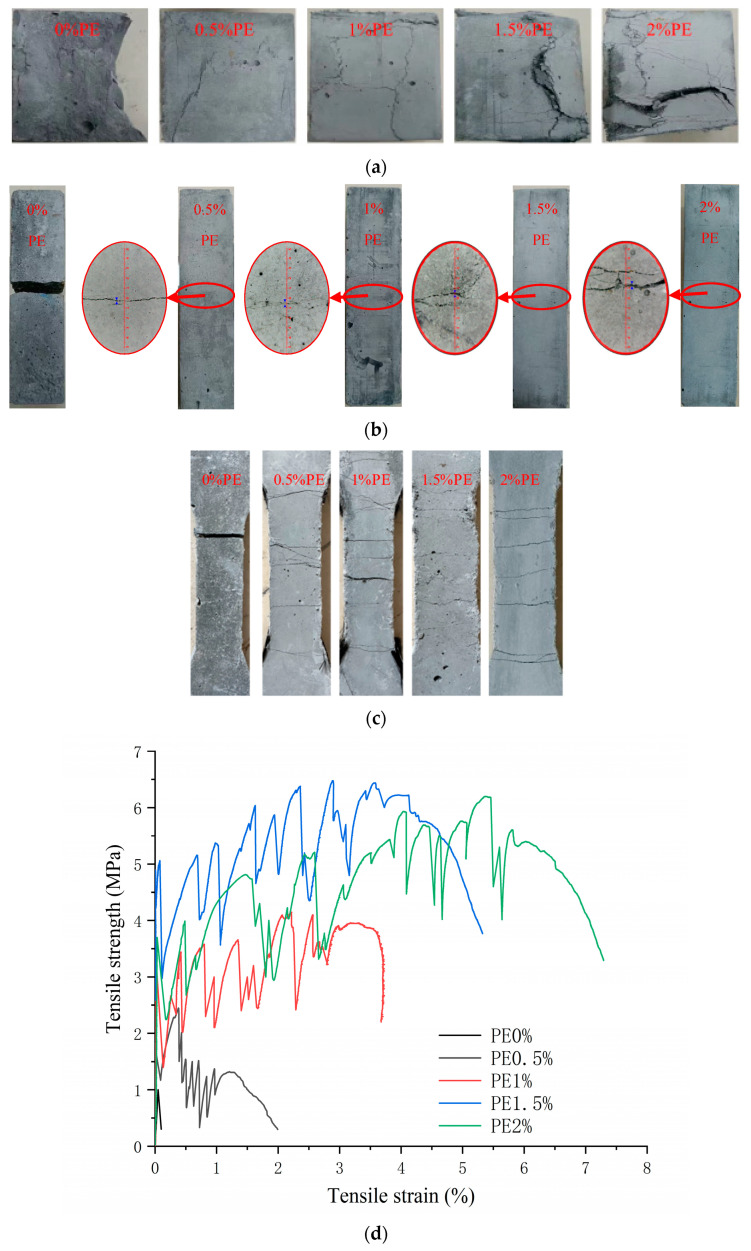
The failure mode of the test block under different PE fibre contents. (**a**) Effect of different fibre contents on compressive failure mode. (**b**) Effect of different fibre contents on flexural failure mode. (**c**) Effect of different fibre contents on tensile failure mode. (**d**) Tensile stress–strain curves of different PE fibre contents.

Figure 4a shows that as the PE fibre content increases, obvious changes in the properties of the matrix appear. When the fibre content is 0.5%, the tensile strength and flexural strength are improved, while the compressive strength is decreased. This phenomenon occurs due to the bridging effect of the fibre, which can delay the generation of macroscopic cracks and improve the tensile and flexural strengths. However, due to the worm effect of the soft fibre, many bubbles are generated when the fibre is introduced. These bubbles are not easy to discharge, and pores are formed after the matrix is formed, reducing the density and compressive strength of the matrix. When the fibre content is 1%, the damage mode of the specimen changes from brittle damage to ductile damage, as shown in Figure 5. This effect occurs because when the content of PE fibre is less than 1%, the fibre bridging ability is weak due to the few fibres, which decreases the level of control when cracking occurs; moreover, the matrix failure mode is brittle failure. As the fibre content continues to increase, the matrix changes to ductile failure that can withstand further loading, and the compressive strength increases [12]. The mechanical properties reach their maximum value at 1.5%. When the fibre content reaches 2%, it begins to decline due to the poor dispersion effects of the fibres in the matrix when too many fibres are added, resulting in an agglomeration effect that reduces the mechanical properties. The tensile stress–strain curve is shown in Figure 5d. When the content of PE fibre reaches 1%, the matrix exhibits strain hardening behaviour when subjected to tension. With the generation and development of matrix cracks, the strength of the matrix increases. The maximum elongation of the matrix increases, increasing the fibre content; however, when the fibre content is more than 1.5%, the elongation growth rate slows. Thus, the optimal value of PE fibre doping is determined to range from 1% to 2%. From Figure 4b,c, the mechanical property indices of CWs and CNTs show trends of first increasing and then decreasing, and the enhancements in the mechanical properties of ECC specimens by CW and CNT micromaterials and nanomaterials are mainly divided into two aspects. The first aspect is the filling effect. Both substances can be used as fillers for microscopic pores in the ECC matrix, improving their gradation by promoting each other and improving the compactness of the matrix in the microscale and nanoscale range, enhancing the mechanical properties. The second aspect is the bridging effect. Both materials are fibrous with exceptionally large aspect ratios, which can produce a bridging effect in ECCs and improve their general mechanical properties by improving the strengths of the interfaces between the CSH gel and quartz sand grains. However, after the doping amounts of CWs and CNTs exceed 2% and 0.15%, respectively, CW agglomerates in the matrix and produces an aggregation effect, which negatively affects the strength of the cementitious material and causes the mechanical properties of the ECC specimens to show a decreasing trend. Therefore, the optimal values of CW doping are in the range of 2% to 4%, and the optimal range of CNT doping is 0.1% to 0.2%.

### 3.2. Analysis of the Microscopic Enhancement Mechanism

**Figure 6:**
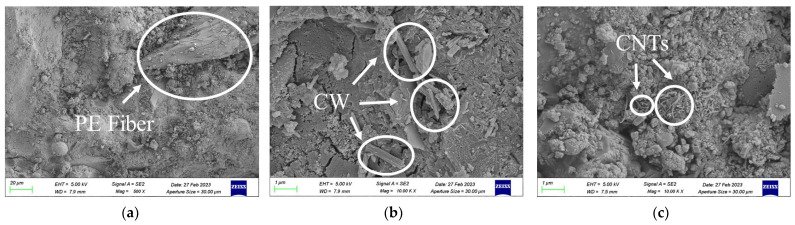
Distribution of PE (**a**), CWs (**b**), and CNTs (**c**) under SEM.

Figure 6a shows that the addition of PE fibres to cement composites creates a fibre-bridging effect. During the tensile process of the material, the PE fibre surface shows obvious scratches and filamentous tears, while the cement hydration products adhere to the fibre surface, making the fibre show moderate slippage and pull-out characteristics. The substantial levels of slip and pull-out increase the crack width and are accompanied by the generation of new cracks, eventually forming a multiple-cracking phenomenon and producing ductile damage.

Figure 6b shows that the CWs are evenly distributed in the matrix and bonded to the cement at both ends, acting as a bridge to support the cementitious system, thereby improving the compactness of the cement matrix, filling the pores, and bridging the microscopic cracks at the microscopic level. The filling effect and bridging effect eventually enhance the mechanical properties and the strain-hardening abilities of HE-ECC.

Figure 6c shows a microscopic photograph of doped carbon nanotubes, and the carbon nanotubes exhibit uniform dispersion in the cementitious material; the nanotubes are interspersed between the cementitious materials and not entangled. According to this analysis, carbon nanotubes act as nucleation sites for hydration products in the matrix of the gelling material and as connections with the loose C-S-H gel, thus forming a dense hydride [41,42,43]. In addition, carbon nanotubes play a bridging role in the matrix, lap at the matrix cracks, and effectively prevent the occurrence and expansion of cracks. The carbon nanotubes form a mesh structure in ultrahigh-performance concrete, thus playing a reinforcing role.

During the tensile failure of ECCs, PE fibres transmit the main stress. At the microscopic level, CWs and CNTs play key roles in the enhancement in the micro/nanostructure; both produce whisker deflection, pull-out, and bridging effects in the matrix, improving the strength of the interfacial transition zone between the fibres and the matrix and increasing the mechanical properties of the ECC. In addition, adding micromaterials and nanomaterials increases the compactness of the matrix, improves the roughness of the PE fibre surface, increases the adhesion between the fibre and matrix, and increases the tensile strength of the ECC.

### 3.3. Response Surface Optimisation Test

#### 3.3.1. Response Surface Test Design and Results

Based on the results of the above single-factor experiment, the response surface method experimental design is conducted using the Box-Behnken design (BBD) method through Design-Expert software. PE fibres, CWs, and CNTs are selected as independent variables, and they are represented by A, B, and C, respectively. The compressive strength, flexural strength, and tensile strength are the response values, which are represented by Y_1_, Y_2,_ and Y_3_, respectively. The experimental results are shown in Table 5.

#### 3.3.2. Response Surface Regression Model Analysis of Compressive Strength

Multiple regressions are fitted to the experimental data in the table using the response surface method. The quadratic polynomial regression equation between the compressive strength (Y_1_) of the ECC matrix and the PE fibre doping (A), CWs (B), and CNTs (C) is established:

Y_1_ = 9.11250 + 15.90350A + 22.94950B + 258.135C − 0.58AB + 10.3AC − 6.6BC − 5.487A^2^ − 4.75925B^2^ − 848.7C^2^ (1)

After establishing the regression equation model, to assess the accuracy of the response surface regression model, analysis of variance (ANOVA) is conducted on the error sources of the above equations; Table 5 shows the results of the analysis of the regression equations. ANOVA of the regression model is mainly used to determine the significance of the model and the significant effect on the response value by the magnitude of the *p* value of the regression model, the misfit term, the linear effect term, the quadratic effect term, the interaction effect term, and the magnitude of the F value of the regression model. When *p* ≤ 0.05, the regression equation is well fitted and reaches a significant level; when *p* < 0.01, the regression equation fits very well and reaches a highly significant level [44,45].

As seen from Table 6, the model has *p* < 0.0001, indicating that the model is extremely significant. The misfit term is not significant (*p* = 0.9917 > 0.05), indicating a relatively good fit of the equation. The coefficient of determination R^2^ = 0.9842, the adjusted R^2^ is close to R^2^, and the difference between the adjusted R^2^ and predicted R^2^ is not more than 0.2, indicating that the actual and predicted values are highly correlated. Therefore, the model can better reflect the relationship between the ECC factors and response values and predict the optimal multiscale fibre fitting ratio. Among the three single factors of PE fibre, CWs, and CNTs, the effect of CWs is very significant, the effects of PE fibres and CNTs are not significant, and the order of the three factors on compressive strength is B > C > A, i.e., CWs > CNTs > PE, as shown by the F value. Additionally, significance evaluations are made using *p* values to determine the degree of influence of the quadratic terms; in terms of the linear interaction between the two factors, the largest interaction is between the CWs and CNTs, followed by the interactions between the PE fibres and CWs and the PE fibres and CNTs.

**Figure 7:**
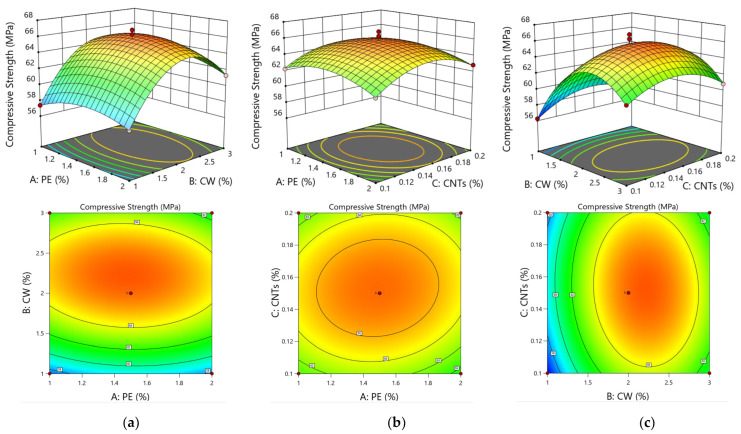
Response surface plots and contour diagram of compressive strength. The interaction of PE-CW (**a**), the interaction of PE-CNTs (**b**), and the interaction of CW-CNTs (**c**).

Figure 7 shows the 3D response surface plot of the interactions between two factors of compressive strength, which can intuitively reflect the degrees of influence and the interactions of PE fibres, CWs, and CNTs on the compressive strength of the ECC matrix.

By combining an analysis of response surface plots and contours, as shown in Figure 7a, it can be concluded that the interactions of CWs and CNTs have the most significant effect on the compressive strength; additionally, the increase in the compressive strengths of ECCs by CWs and CNTs at the microscale and nanoscale are increasingly obvious. However, due to the reduced doping of CNTs and a highly complicated dispersion method, the effect is limited relative to CWs, and the conclusion is consistent with the results obtained from the model regression coefficients. Figure 7b shows the 3D response surface of the effect of the interaction of PE fibres and CNTs on the compressive strength. When the CNT content is 0.15%, the doping of PE fibres and CWs either increases or decreases, and the compressive strength fluctuates by 2 MPa. This conclusion is consistent with the results obtained from the model regression coefficient *p* value, which shows that the interaction is not very significant. This phenomenon occurs because, among the compressive damage, the millimetre-scale PE fibres mainly change the damage morphology, and the ECC specimens are converted from brittle to ductile damage, which does not significantly improve the compressive strength. As shown in Figure 7c, when the CW content is 1%, the CNT content increases from 0.1% to 0.2% and the compressive strength increases from 56.26 MPa to 57.33 MPa; when the CW content is 3%, the CNT content increases from 0.1% to 0.2% and the compressive strength decreases from 61.1 MPa to 60.85 MPa. When both materials are dosed too high, the dispersion effect worsens, and the agglomeration effect is increased. The decrease in compressive strength is highly obvious. In summary, among the three factors of PE fibres, CWs, and CNTs, the effects of CWs and CNTs on the compressive strengths of ECCs are the most obvious, and the interaction between them is the most significant.

#### 3.3.3. Response Surface Regression Model Analysis of Flexural Strength

Multiple regressions are fitted to the experimental data in the table using the response surface methodology. The quadratic polynomial regression equation is established between the 28 d flexural strength (Y_2_) of the ECC matrix and the doped PE fibres (A), CWs (B), and CNTs (C):

Y_2_ = −19.08 + 25.65650A + 5.298B + 87.115C − 0.32AB − 6.1AC − 1.3BC − 7.613A^2^ − 1.06575B^2^ − 241.3C^2^ (2)

As seen from Table 7, the model has a *p* value of *p* < 0.0001, indicating that the model is extremely significant. The misfit term is not significant (*p* = 0.7230 > 0.05), indicating a relatively good fit of the equation. The coefficient of determination is R^2^ = 0.9953, the adjusted R^2^ is close to R^2^, and the difference between the adjusted R^2^ and the predicted R^2^ is at most 0.2, indicating that the actual and predicted values are highly correlated. Therefore, the model can effectively reflect the relationship between the ECC factors and the response value flexural strength, and it can predict the optimal multiscale fibre fit ratio. Among the three single factors of PE fibres, CWs, and CNTs, the effects of PE fibres are very significant, the effects of CWs are significant, and the effects of CNTs are not significant. The order of the three factors on compressive strength is A > B > C, i.e., PE fibres > CWs > CNTs, as shown by the F value.

**Figure 8:**
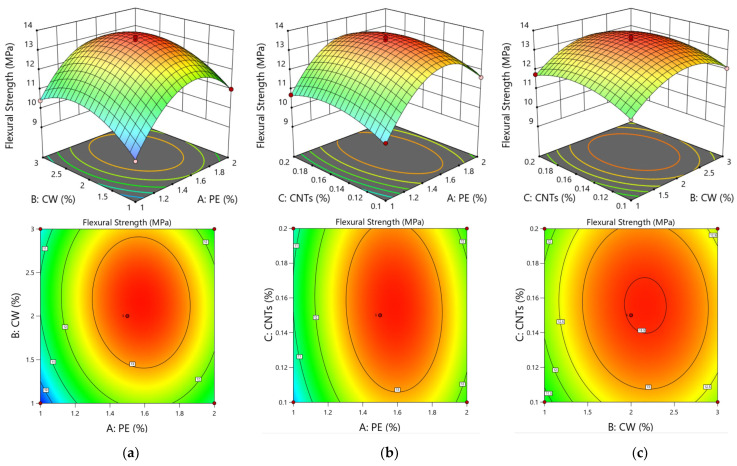
Response surface plots and contour diagram of flexural strength. The interaction of PE-CWs (**a**), the interaction of PE-CNTs (**b**), and the interaction of CW-CNTs (**c**).

Figure 8 shows the 3D response surface plots of the interactions of two factors for flexural strength. The plot shows the effects of the interactions of the remaining two factors on the 28 d flexural strength of the ECC matrix when the third factor is coded as 0 at the medium level.

As shown in Figure 8, the integrated response surface plots and contours are analysed, and the interactions between PE fibres and CWs and between PE fibres and CNTs are the most obvious. As shown in Figure 8a, when the CW content is 2%, the flexural strength of the matrix tends to first increase and then decrease with increasing PE fibre doping, and the overall fluctuation is large. When the PE fibre admixture is approximately 16%, the flexural strength reaches its maximum value. As shown in Figure 8b, when the CNTs are at a doping level of 0.14, the fluctuations increase with the increase in the doping level of PE fibres. When interacting with PE fibres, the effect on flexural strength is highly pronounced, which is consistent with the results obtained from the regression coefficient *p* of the above model. As shown in Figure 8c, the fluctuations in the 3D response surface of the interaction between CWs and CNTs on the impact of flexural strength are small, indicating that the impact on flexural strength does not change regardless of the increase or decrease in CW or CNT doping; thus, the interaction is not significant, which is consistent with the results of the *p* value of the regression coefficient of the above model.

This finding shows that in the matrix flexural experiment, fibres can effectively inhibit the development of macroscopic cracks due to their large size. The role of fibres in bridging cracks is the main factor. Due to its filling effect and bridging effect, CWs and CNTs increase the compactness of the matrix and reduce the generation of microcracks inside the matrix; CWs improve the flexural properties of the matrix through whisker pull-out, crack deflection, and crack bridging, thereby reducing the generation and development of microcracks [45,46,47]. CNTs strengthen the interfacial transition zone by filling nanopores and bridging cracks, resulting in a dense microstructure and high crack resistance to fibre pullout. He proposed the use of CNTs to enhance the interfacial transition zone and interfacial friction bonding strength between PE fibres and cement-based matrices. The small CNTs can be attached to the surface of the PE fibre, which can improve the bonding force between the PE fibre and matrix to improve flexural strength [48].

#### 3.3.4. Response Surface Regression Model Analysis of Axial Tensile Strength

Multiple regressions are fitted to the experimental data in the table using the response surface method. A quadratic polynomial regression equation is established between the tensile strength (Y_3_) of the ECC matrix and the PE fibre doping (A), CWs (B), and CNTs (C):

Y_3_ = −17.43375 + 25.1625A + 3.44375B + 22.05C − 0.115AB − 1.8AC − 0.6BC − 7.975A^2^ − 0.75875B^2^ − 58.5C^2^ (3)

As seen from Table 8, the model has a *p* value of *p* < 0.0001, indicating that the model is extremely significant. The misfit term is not significant (*p* = 0.7357 > 0.05), indicating a relatively good fit of the equation. The coefficient of determination is R^2^ = 0.9973, the adjusted R^2^ is close to R^2^, and the difference between the adjusted R^2^ and predicted R^2^ is at most 0.2, indicating that the actual and predicted values are highly correlated. Therefore, the model can effectively reflect the relationship between the ECC factors and response values, and it can predict the optimal multiscale fibre fitting ratio. Among the three single factors of PE fibres, CWs, and CNTs, the effects of PE fibres are very significant, the effects of CWs are significant, and the effects of CNTs are not significant. According to the *p* value, the order of the three factors on compressive strength is A > B > C, i.e., PE fibres > CWs > CNTs.

**Figure 9:**
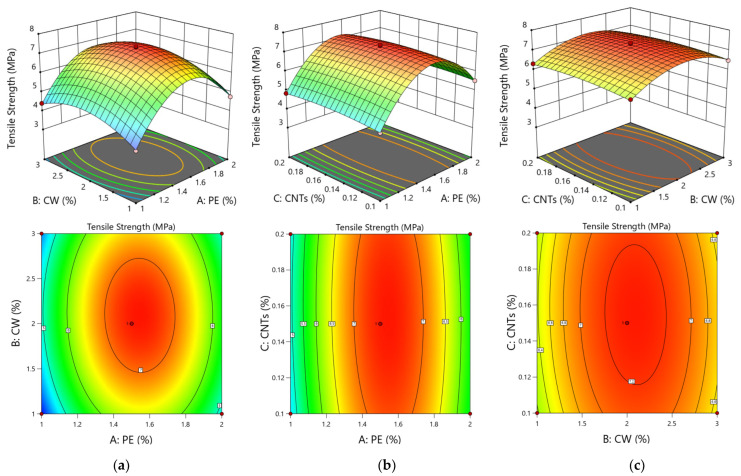
Response surface plots and contour diagram of tensile strength. The interaction of PE-CW (**a**), the interaction of PE-CNTs (**b**), and the interaction of CW-CNTs (**c**).

Comprehensive response surface plots and contours are analysed. As shown in Figure 9a, the interaction of the PE fibres and CWs has a significant effect on tensile strength. When the doped amount of CWs is certain, the tensile strength of the ECC matrix first increases and then decreases with increasing PE fibre content. When the doped amount of PE fibres is certain, the tensile strength of the ECC matrix shows a trend of first increasing and then decreasing with increasing CW content, which is consistent with the *p* value results of the regression coefficient AB of the table model. As seen in Figure 9b, the tensile strength of the matrix does not change substantially with the increase in the doped amount of CNTs when the doped amount of PE fibres is certain; the interaction of the two factors does not have a significant effect on the flexural strength of the ECC matrix. As seen in Figure 9c, the 3D response surface of the effect of the interaction of CWs and CNTs on the compressive strength is approximately planar, indicating that the tensile strength is almost unaffected regardless of the increase or decrease in the doped amounts of CWs and CNTs; additionally, the interactions of the two factors on the response value are not significant, which has the same effect as the 28 d tensile strength of the ECC substrate. In summary, among the three factors of PE fibres, CWs, and CNTs, PE fibres most significantly affect the tensile strengths of the ECC, and the interaction between PE fibres and CWs is the most obvious.

In the tensile test, the tensile bearing capacity of the ECC matrix is mainly derived from the strengthening and toughening mechanisms of the PE fibre. After cracks occur in the specimen, the fibre bridge embedded at both ends of the matrix plays a role in stress transfer to the matrix. The CWs exhibit whisker pull-out and deflection and absorb the energy transmitted by the external load through deformation. CNTs can bridge cracks and react with OH groups in the hydration reaction to improve the chemical bonding force [49]. However, due to the small size and content of CNTs, the effects of CNTs in this study are not obvious.

### 3.4. Prediction and Verification of Optimal Process Conditions

The experimental data are optimally predicted using Design-Expert 11, and multiobjective optimisation of PE fibres, CWs, and CNTs is performed with maximum compressive strength, flexural strength, and tensile strength as the optimisation objectives to obtain the optimal mix ratio of multiscale-reinforced ECCs. Table 8 shows the optimised best fit ratio and a comparison of experimental predictions and values, and the optimisation results are verified using the absolute value of the relative error D. The calculation formula is as follows:

(4) $$D=\frac{\left|Y_{T}-Y_{P}\right|}{Y_{T}}\times 100\%$$

where $Y_{T}$ is the measured value and $Y_{P}$ is the predicted value.

**Figure 10:**
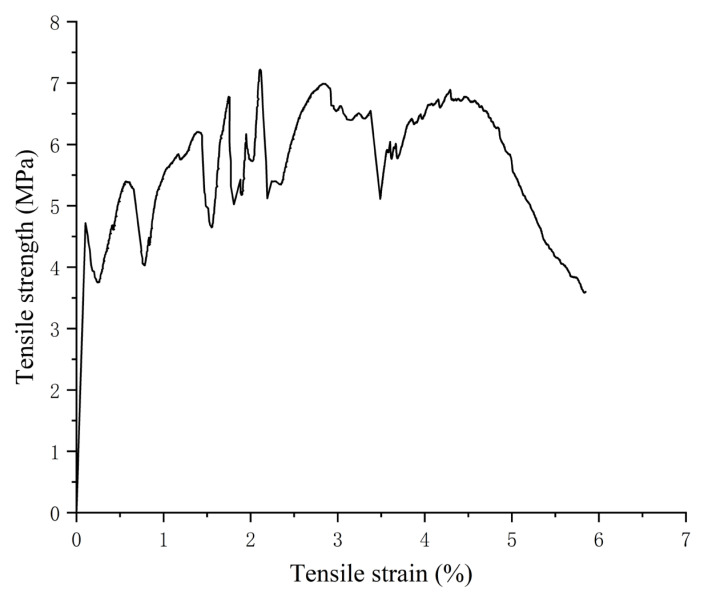
Tensile stress–strain curve of the final optimisation results.

As seen from Table 9, the absolute values of the relative errors between the predicted values of compressive strength, flexural strength, and tensile strength and the experimental values are 1.69%, 3.03%, and 2.8%, respectively; these values are all less than 5%, indicating that the established prediction models of compressive strength, flexural strength, and tensile strength of the ECC matrix have high accuracy and are representative. In the tensile test, the final optimisation results still show the strain hardening phenomenon and the elongation exceeds 3%, as shown in Figure 10. This result shows that the final optimisation results of this paper meet the design concept of ECCs and can be applied in ECCs.

As shown in Table 9, after using the final optimised mix proportion of this paper, the experimental results show that the compressive strength reaches 67.1 MPa, the flexural strength reaches 13.2 MPa, and the tensile strength reaches 7.1 MPa. Compared with other studies, Wu [32] used a hybrid of CWs and a partial substitution of PE fibres; its maximum compressive strength does not exceed 50 MPa. Shokrieh [50] used a single-doped PVA fibre to obtain a maximum flexural strength of less than 3.5 MPa. Pan [25] designed a new multiscale ECC composed of PVA fibres and CW; its maximum tensile strength does not exceed 4 MPa. In summary, based on the multiscale fibre content, the final optimised mix proportion in this paper can significantly improve the mechanical properties of ECCs.

## 4. Conclusions

(1) Through the single-factor experiment, it can be concluded that when the PE fibre content is more than 1%, the ECC matrix exhibits strain hardening properties in tensile tests, thus showing increased strength. With additions of CWs and CNTs, the strength of the ECC matrix first increases and then decreases. This phenomenon occurs because when the content of CWs exceeds 3% and that of CNTs exceeds 0.2%, agglomeration occurs, which reduces the mechanical properties of the ECC matrix. When the content of PE fibres is between 1% and 2%, the content of CWs is between 1% and 3%, and the content of CNTs is between 0.1% and 0.2%, the ECC matrix has the best mechanical properties. In addition, the water–binder ratio, fibre type, and fibre length affect the mechanical properties of ECCs. In this paper, other influencing factors are not studied, due to the limited space.
(2) Among the three factors affecting the mechanical properties of the ECC matrix, the significant order of influence on the compressive strength of the ECC matrix is as follows: CWs, CNTs, and PE fibres. Additionally, the order of influence on the flexural and tensile strengths of the ECC matrix is as follows: PE fibres, CWs, and CNTs. This finding shows that the PE fibres have the most obvious effects on the flexural and tensile strengths, and the CWs and CNTs have the most obvious effects on the compressive strength.
(3) When the doping level of PE fibres is 1.55%, that of CWs is 2.17%, and that of CNTs is 0.154%, the response surface model predicts the compressive strength of the ECC matrix as 65.964 MPa. The actual test value is 67.1 MPa, the flexural strength is 13.601 MPa, the actual test value is 13.2 MPa, and the tensile strength is 7.299 MPa. The actual test value is 7.1 MPa. The absolute value of the relative error between the predicted and tested values is less than 5%, which proves that the use of the response surface method can achieve the multiobjective optimisation of the ECC mix proportion.
(4) Finally, in this experimental design, the optimised test results show that the optimised mix ratio is 1.55% PE fibres, 2.17% CWs, and 0.154% CNTs, which can significantly improve the comprehensive mechanical properties of ECCs. The test results are accurate and reliable, thereby providing a reference for the design of the ECC mix ratio. Note that this mix design scheme only involves the multiscale fibre content ratio, not other influencing factors.

## Figures and Tables

**Table 1:** Chemical composition of cement and fly ash.

	SiO_2_	Al_2_O_3_	CaO	Fe_2_O_3_	Na_2_O	MgO	K_2_O	TiO_2_	SO_3_
Cement	21.89	5.58	62.55	2.86	0.83	2.26	0.87	0.19	2.89
Fly ash	48.42	29.93	7.56	6.83	1.32	1.27	1.24	1.72	4.65

**Table 2:** Physical properties of different scales fibres.

Fiber Type	Length (μm)	Aspect Ratio	Density (g/cm^3^)
PE	12,000	600	0.97
CW	50–80	13	2.8
CNTs	5–15	200–400	1.8

**Table 3:** Single-factor experiment mix proportion.

Mixture	Cement	Sand	Fly Ash	PE Fibers (%)	CW (%)	CNTs (%)	Water	Superplasticiser
PE0	1350	1350	1350	0	2	0.1	810	27
PE0.5				0.5	2	0.1		
PE1				1	2	0.1		
PE1.5				1.5	2	0.1		
PE2				2	2	0.1		
CW0				1.5	0	0.1		
CW1				1.5	1	0.1		
CW2				1.5	2	0.1		
CW3				1.5	3	0.1		
CW4				1.5	4	0.1		
CNTs0				1.5	2	0		
CNTs0.05				1.5	2	0.05		
CNTs0.1				1.5	2	0.1		
CNTs0.15				1.5	2	0.15		
CNTs0.2				1.5	2	0.2		

**Table 4:** Codes and levels of experimental independent variable factors.

Level	Factors		
	A: PE	B: CW	C: CNTs
−1	1	1	0.1
0	1.5	2	0.15
1	2	3	0.2

**Table 5:** Test design and results.

Test Group Number	A	B	C	Compressive Strength (MPa)	Flexural Strength (MPa)	Tensile Strength (MPa)
1	−1	−1	0	57.38	9.33	3.92
2	1	−1	0	57.74	11.02	4.76
3	−1	1	0	62.11	10.41	4.41
4	1	1	0	61.31	11.46	5.02
5	−1	0	−1	62.22	10.17	4.68
6	1	0	−1	61.58	11.63	5.52
7	−1	0	1	62.45	10.71	4.85
8	1	0	1	62.84	11.56	5.51
9	0	−1	−1	56.26	11.27	6.22
10	0	1	−1	61.1	12.08	6.49
11	0	−1	1	57.33	11.76	6.32
12	0	1	1	60.85	12.31	6.47
13	0	0	0	65.54	13.32	7.32
14	0	0	0	64.89	13.53	7.11
15	0	0	0	66.87	13.68	7.25
16	0	0	0	66.32	13.42	7.35
17	0	0	0	65.21	13.67	7.37

**Table 6:** Variance analysis of compressive strength regression model.

Source	Sum of Squares	df	Mean Square	F-Value	*p*-Value	Significance
Model	168.62	9	18.74	48.32	<0.0001	※※
A-Polyethylene Fiber	0.0595	1	0.0595	0.1535	0.7069	
B-Calcium Carbonate Whisker	34.69	1	34.69	89.47	<0.0001	※※
C-Carbon Nanotube	0.667	1	0.667	1.72	0.2311	
AB	0.3364	1	0.3364	0.8675	0.3826	
AC	0.2652	1	0.2652	0.684	0.4355	
BC	0.4356	1	0.4356	1.12	0.3244	
A^2^	7.92	1	7.92	20.43	0.0027	※
B^2^	95.37	1	95.37	245.95	<0.0001	※※
C^2^	18.96	1	18.96	48.88	0.0002	※
Residual	2.71	7	0.3878			
Lack of Fit	0.0611	3	0.0204	0.0307	0.9917	
Pure Error	168.62	9				
Cor Total	0.0595	1				
R^2^	0.9842					
Adjusted R^2^	0.9638					
Predicted R^2^	0.9701					

※※ means very significant, ※ means significant.

**Table 7:** Variance analysis of flexural strength regression model.

Source	Sum of Squares	df	Mean Square	F-Value	*p*-Value	Significance
Model	28.1	9	3.12	165.29	<0.0001	※※
A-Polyethylene Fiber	3.19	1	3.19	168.74	<0.0001	※※
B-Calcium Carbonate Whisker	1.04	1	1.04	54.88	0.0001	※
C-Carbon Nanotube	0.177	1	0.177	9.37	0.0183	※
AB	0.1024	1	0.1024	5.42	0.0528	
AC	0.093	1	0.093	4.92	0.062	
BC	0.0169	1	0.0169	0.8946	0.3757	
A^2^	15.25	1	15.25	807.32	<0.0001	※※
B^2^	4.78	1	4.78	253.14	<0.0001	※※
C^2^	1.53	1	1.53	81.11	<0.0001	※※
Residual	0.1322	7	0.0189			
Lack of Fit	0.0341	3	0.0114	0.4637	0.723	
Pure Error	0.0981	4	0.0245			
Cor Total	28.24	16				
R^2^	0.9953					
Adjusted R^2^	0.9893					
Predicted R^2^	0.9752					

※※ means very significant, ※ means significant.

**Table 8:** Variance analysis of tensile strength regression model.

Source	Sum of Squares	df	Mean Square	F-Value	*p*-Value	Significance
Model	21.56	9	2.4	283.57	<0.0001	※※
A-Polyethylene Fiber	1.09	1	1.09	128.79	<0.0001	※※
B-Calcium Carbonate Whisker	0.1711	1	0.1711	20.26	0.0028	※
C-Carbon Nanotube	0.0072	1	0.0072	0.8524	0.3866	
AB	0.0132	1	0.0132	1.57	0.251	
AC	0.0081	1	0.0081	0.959	0.3601	
BC	0.0036	1	0.0036	0.4262	0.5347	
A^2^	16.74	1	16.74	1981.55	<0.0001	※※
B^2^	2.42	1	2.42	286.99	<0.0001	※※
C^2^	0.0901	1	0.0901	10.66	0.0138	※
Residual	0.0591	7	0.0084			
Lack of Fit	0.0147	3	0.0049	0.4422	0.7357	
Pure Error	0.0444	4	0.0111			
Cor Total	21.62	16				
R^2^	0.9973					
Adjusted R^2^	0.9937					
Predicted R^2^	0.9859					

※※ means very significant, ※ means significant.

**Table 9:** Comparison of predicted and actual values after ratio optimisation.

PE (%)	CW (%)	CNTs (%)	Strength (MPa)	Predicted Value	Experimental Value	D%
1.549	2.172	0.154	Compressive Strength	65.963	67.1	1.69
			Flexural Strength	13.601	13.2	3.03
			Tensile Strength	7.299	7.1	2.8

## Data Availability

The data used to support the findings of this study are available from the corresponding author upon request.
